# The foot plantar pressures for patients with hallux valgus combines with or without claw toe

**DOI:** 10.1186/1757-1146-7-S1-A110

**Published:** 2014-04-08

**Authors:** Wen-Lan Wu, Jina-Min Liang, Yuh-Min Cheng, Peng-Ju Huang, Jia-Hroung Wu

**Affiliations:** 1 Department of Sports Medicine, Kaohsiung Medical University, Kaohsiung, Taiwan; 2 Department of Orthopaedic Surgery, Kaohsiung Medical University Hospital, Kaohsiung, Taiwan; 3 Department of Industrial Engineering & Management, Hsiuping University of Science and Technology, Taiwan

## Background

Hallux valgus and claw toe remains the most common and disabling pathologies of the foot. The goal was to assess the difference between the patients suffered hallux valgus combine with claw toe and the patients without any complications.

## Methods

7 severe HV patients (2 male and 5 female, 14 foot) and 7 patients suffered HV combine with claw toe (HVC) (1 male and 6 female, 14 foot) were recruited in this study. The F-Scan in-shoe system (Tekscan, Inc.) were used to record the data of plantar pressure at a sampling frequency of 50 Hz. Subjects were instructed to wear the custom shoes with F-Scan sensor pad and perform a 5 meter walking task on their self speed. For plantar pressure assessment, each footprint is divided into 12 regions ( MH, LH, MF, M1, M2, M3, M4, M5, T1, T2, T3, T45) to enable analysis of the instant of peak pressure (kg/cm2) and maximum force that were normalized with body weight (MxF(%BW)). The independent t test was used to determine the differences of foot regions for two groups. Results were considered statistically significant when the p<0.05.

## Results

The result of anthropometric feature of the individuals recruited for the study was showed in Table 1. It showed that HVC group had significantly larger degree between M1and M2. The Table 2 showed the MxF (%BW) and peak pressure (kg/cm2) that had significant difference from two groups. It showed that the HVC group had lower PP and MxF than HV group at the T1 region; conversely, it showed higher MxF at the M2 and M3 regions.

**Table 1:** Anthropometric feature of the individuals recruited for the study (mean ± SD)

	HV	HVC
Age (year) ^#^	38.42±13.54	67.86±9.14
Height (cm)	165.29±9.72	159.14±6.20
Weight (kg)	63.86±10.17	65.28±4.89
Degree of HV (°)	33.26±7.81	38.72±10.68
Degree of M1M2 (°) ^#^	12.28±2.08	15.61±3.39
Foot width (mm)	91.29±9.56	95.84±4.23

# means p<0.05

**Table 2:** Peak pressure and MxF(%BW) for walking are expressed as mean ± SD.

	HV	HVC
Peak pressure (kg/cm^2^)		
M2	4.45±1.91	5.78±3.36
M3	3.64±1.60	4.73±1.57
T1^#^	4.06±2.07	2.74±1.60
MxF (%BW)		
M2^#^	24.58±8.48	32.16±11.29
M3^#^	19.92±5.69	25.12±7.17
T1^#^	23.74±15.02	11.41±7.29

# means p<0.05

## Conclusion

In past study, Plank found a medial shift in peak pressure in most of the hallux valgus group, with a significant decrease in pressure beneath the fourth and fifth metatarsal heads and this finding was also be found from HV and HVC group. In the present study, when patients suffered HV combined with claw toe represented high peak pressure and MxF at the M2and M3 regions. It would result in the metatarsal heads bear more weight and become painful during walking.
